# Antibiotic treatment of acute gastroenteritis in children

**DOI:** 10.12688/f1000research.12328.1

**Published:** 2018-02-15

**Authors:** Eugenia Bruzzese, Antonietta Giannattasio, Alfredo Guarino

**Affiliations:** 1 Department of Translational Medical Sciences–Section of Pediatrics, University of Naples Federico II, Via S. Pansini 5, Naples, 80131, Italy

**Keywords:** gastroenteritis, diarrhoea, vomiting, antimicrobials, children

## Abstract

Antibiotic therapy is not necessary for acute diarrhea in children, as rehydration is the key treatment and symptoms resolve generally without specific therapy. Searching for the etiology of gastroenteritis is not usually needed; however, it may be necessary if antimicrobial treatment is considered. The latter is left to the physician evaluation in the absence of clear indications. Antimicrobial treatment should be considered in severely sick children, in those who have chronic conditions or specific risk factors or in specific settings. Traveler’s diarrhea, prolonged diarrhea, and antibiotic-associated diarrhea may also require antibiotic therapy. Depending on the severity of symptoms or based on risk of spreading, empiric therapy may be started while awaiting the results of microbiological investigations. The choice of antibiotic depends on suspected agents, host conditions, and local epidemiology. In most cases, empiric therapy should be started while awaiting such results. Empiric therapy may be started with oral co-trimoxazole or metronidazole, but in severe cases parenteral treatment with ceftriaxone or ciprofloxacin might be considered.

## Introduction

Acute gastroenteritis (AGE) is one of the most common problems in infants and young children, especially in poor countries. It is caused by viral, bacterial, and parasitic agents, with an age-, host-, and location-based pattern. Etiology usually is not looked for, and oral rehydration therapy is the universal therapy. Active treatment with probiotics and antidiarrheal agents is suggested in adjunct to rehydration, as it reduces the duration and intensity of symptoms independently from etiology ^1^. There are no clear indications for antimicrobial therapy; however, antibiotics are frequently prescribed. Overuse of antibiotics is associated with increased rates of antibiotic-resistant bacteria, unnecessary costs, and significant incidence of adverse events, and current guidelines are highly restrictive in recommending empiric antimicrobial therapy for AGE. Bacterial infections may be associated with the presence of specific clinical features, notably fever, abdominal pain, blood in the stool, and fecal leukocytes ^2^. However, none of these features is reliable to support a bacterial etiology. In addition, many children with bacterial enteritis have negative stool cultures and, conversely, it is not uncommon to detect multiple bacterial and viral pathogens, making it difficult to give a causative role to a specific microorganism.

The application of a quantitative molecular approach showed that four agents (rotavirus, *Cryptosporidium*, enterotoxigenic *Escherichia coli* (ETEC) producing heat-stable toxin, and *Shigella*) account for the majority of cases of infectious diarrhea in African and Asian children younger than 5 years old ^3^. It is a logical hypothesis that, if bacteria are causing gastroenteritis, antibiotic therapy could be effective in reducing the intensity and duration of symptoms and prevent infection spreading. Furthermore, specific antibiotic treatment may prevent serious complications such as sepsis and protracted diarrhea in children with underlying conditions such as immunosuppression or malnutrition. However, the indications for antibiotic therapy are not standardized, and randomized controlled trials are not available in children.

## Bacterial etiology of acute gastroenteritis in developing and developed countries

The etiological pattern of bacteria causing acute diarrhea depends on geographical area. In developing countries, more than half a million infants and young children die each year because of AGE, and *Vibrio cholerae* still causes epidemics, but the most common bacterial agent is *Shigella* ^4^. In Europe, the most common bacterial pathogens are *Campylobacter*, *Salmonella* spp., enteropathogenic *E. coli* (EPEC), and enteroaggregative *E. coli* (EAEC) ^5, 6^. *Clostridium difficile* (Cd) has emerged as a cause of community-acquired diarrheal illness, but local data report a relatively low burden ^7– 9^. In Ecuador, sub-Saharan Africa, and South Asia, *Shigella* is the main agent ^3, 10^. In a recent study from central China, pathogens were detected in 20% of 508 fecal samples from patients with acute diarrhea, under 5 years of age ^11^. The most commonly detected pathogens were *Salmonella* spp. (8%), diarrheagenic *E. coli* (5%), *Campylobacter jejuni* (3%), and *Aeromonas* spp. (2%). In the developing region of China, *Shigella* was the most common bacterial agent of AGE ^12^. In India, *E. coli* was the most common agent of AGE (31%) followed by *Shigella* (24%). Infections with two or more pathogens were observed in 34% of cases, with a predominant incidence in children younger than 2 years old ^13^.

Bacterial pathogens account for 80% of cases of traveler’s diarrhea ^14^. ETEC, enteroinvasive *E. coli* (EIEC), and EAEC are implicated in the majority of cases, but also *Campylobacter*, *Salmonella*, and *Shigella* play a substantial role.

## Current recommendations for the treatment of acute gastroenteritis

Evidence-based indications for the management of children with AGE are that oral rehydration with hypo-osmolar solution is the key treatment and should be started as soon as possible ^1^. The so-called active intervention in adjunct to rehydration includes specific probiotics such as *Lactobacillus rhamnosus* strain GG or *Saccharomyces boulardii*, or diosmectite or racecadotril. Active treatment reduces the intensity of symptoms and their duration independently of etiology ^15^. However, the concept of active treatment of gastroenteritis is progressively pursued in children, and current recommendations for the use of probiotics and antidiarrheal drugs are available from several regions of the world, including the Asia-Pacific region ^15^. According to the guidelines for the management of AGE, antibiotic therapy should not be given to the vast majority of children with AGE, unless specific conditions are present. Even in cases of proven bacterial gastroenteritis, antibiotic therapy is not routinely needed but should be considered only for specific pathogens or in defined clinical settings.

The routine use of antimicrobials for diarrhea in children is not recommended by the World Health Organization (WHO) except for clinically recognizable severe cases ^16^. It is indicated in the following circumstances: cholera, shigellosis, dysenteric presentation of campylobacteriosis and non-typhoidal salmonellosis when they cause persistent diarrhea, and when host immune status is compromised for any reason including severe malnutrition, chronic disease, or lymphoproliferative disorders. Antimicrobial treatment should also be considered for: moderate/severe traveler’s diarrhea or diarrhea accompanied by fever and/or bloody stools and diarrhea associated with another acute infection (e.g. pneumonia) requiring specific antimicrobial therapy. Similar indications are provided at a local level, but supporting evidence is weak or absent ^17, 18^.

## Antimicrobial prescribing patterns for acute gastroenteritis in developing and developed countries

Antibiotic therapy is sometimes recommended to shorten the duration and severity of symptoms of AGE as well as to decrease its transmission ^19, 20^. The emerging challenge of antibiotic resistance complicates treatment for bacterial diarrhea. Antimicrobial resistance among diarrheal pathogens is high in developing countries, where the use of antimicrobials is less restricted, and these rates are on the rise worldwide ^21, 22^.

In developing countries, guidelines for acute diarrhea suggest that the presence of blood in the stools should always be checked. Non-bloody diarrhea should be managed with fluids only (unless co-morbidities are present that may require a different treatment), while dysentery (reported history of blood in the stools since diarrheal onset) should be managed with antibiotics, as *Shigella* infection is suspected ^23^. This approach is supported by the evidence that most non-bloody diarrheal episodes in children under 5 years of age in low-income settings are self-limiting and are caused by viral pathogens (rotavirus, norovirus, astrovirus, and enteric adenovirus) or pathogens for which antibiotics are likely of limited efficacy or even dangerous (e.g. *Salmonellae* and *Campylobacter*) ^24^. In contrast, a significant proportion of episodes of bloody diarrhea caused by *Shigella* is associated with considerable mortality and should be treated with antibiotic therapy ^25^. However, inappropriate antibiotic use remains common. In a study in 447 Indian children aged between 6 months and 5 years, deviations from WHO protocol for AGE treatment were found in 78% of cases ^26, 27^. Although in all cases oral rehydration solution and zinc were prescribed, unnecessary antibiotic use was reported in 12% of cases, with cefixime, ofloxacin, and ceftriaxone being the most frequently prescribed antibiotics. Hospitalization, longer duration of symptoms prior to presentation, and fever were associated with prescription of antibiotics ^27^. In other studies, the type of physician was related to antibiotic prescription. Pediatricians working in the government sector prescribed antibiotics to only 23% of children, while private practitioners prescribed antibiotics to 51% of children with diarrhea ^28^.

Also, in developed countries, over-prescription of antibiotics for AGE was reported, and physician responses to patients’ treatment expectations was an important cause of inappropriate antibiotic use ^29^. However, in as many as 10% of children admitted to hospital, unnecessary antimicrobial therapy is prescribed because of a “probable bacterial cause” ^30^.

## Indications for antimicrobial treatment of acute gastroenteritis

In adults, single cases of acute febrile bloody diarrhea are more likely to be caused by bacterial pathogens such as *Campylobacter* or *Shigella* species, depending on the epidemiological setting. These patients are likely to benefit from empirical antimicrobial therapy ^31^.

In children, there are no clear or validated criteria for antibiotic therapy. However, the criteria for considering antibiotic treatment include clinical features, host-related and setting-related conditions, and, of course, etiology.

Because the etiology of diarrhea is not generally looked for, the decision to treat children with AGE with antibiotics should be based on the presence of factors that “may require” antibiotic treatment (see Table 1 and Table 2). Generally, antibiotic choice should be initially empiric and subsequently tailored on the results of microbiological investigations. In many conditions, waiting for microbiological results to confirm the decision to treat and select the specific drug may be appropriate.

**Table 1.** Clinical conditions and circumstances that may indicate antibiotic therapy.

Condition	Putative bacterial agent	Suggested antibiotic
Dysenteric diarrhea	*Shigella, Yersinia, Campylobacter*	Azithromycin, ciprofloxacin
Fever, increased inflammation markers	*Shigella*	Azithromycin, ceftriaxone
Prolonged diarrhea	Gram-negative enterobacteria, *Clostridium difficile*	Metronidazole, co-trimoxazole
SIBO	Gram-negative enterobacteria	Metronidazole, rifaximin, co-trimoxazole
Antibiotic-associated diarrhea	*Clostridium difficile*, others	Metronidazole, vancomycin (only if *Clostridium difficile* is detected)
Traveler’s diarrhea	ETEC, EPEC	Azithromycin, ciprofloxacin
Toxic state	Gram-negative enterobacteria, *Clostridium difficile*	Ceftriaxone

EPEC, enteropathogenic *Escherichia coli*; ETEC, enterotoxigenic *Escherichia coli*; SIBO, small intestinal bacterial overgrowth.

**Table 2.** Risk factors indicating antibiotic therapy in children with acute diarrhea.

Risk factors	Evidence
***Host-related risk factors***	
Age <3 (or 6) months	Poor evidence but strong indication in neonates
Severity of clinical presentation	Poor evidence but strong indications
Malnutrition	Strong evidence
Chronic underlying disease Immune deficiency	Strong evidence for children with IBD or HIV Oncologic patients in immunosuppression therapy
***Setting-related risk factors***	
Day-care centers, hospitals, Iand close institutions	Strong evidence, if spreading of bacterial infection is an issue
Traveler’s diarrhea	Strong evidence in adults, poor evidence in children

HIV, human immunodeficiency virus; IBD, inflammatory bowel disease.

### Clinical indications

**Figure 1.**
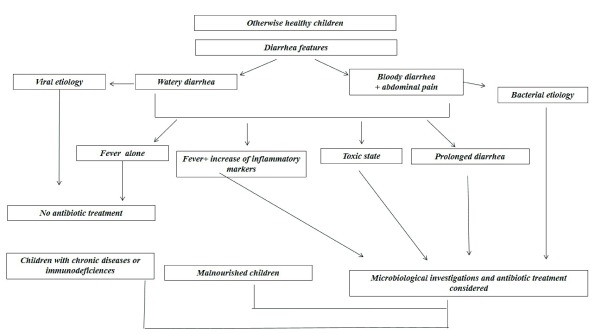
Criteria to decide antibiotic treatment in children with infectious diarrhea.

The guidelines for the treatment of acute diarrhea in children state that the use of antibiotics is not needed routinely but only for specific pathogens or in defined clinical settings ^1^. Clinical indications for antibiotic therapy include toxic state or signs of invasive infection ( Table 1). These should be considered as strong indications to parenteral antibiotic treatment. Fever per se does not require antimicrobial therapy but needs to be considered in a more global clinical evaluation. It may indicate dehydration but also spreading of intestinal infection. This could be confirmed by an increase of inflammatory markers such as C-reactive protein. Dysentery presentation with abdominal pain and mucoid or bloody stools (often in multiple outputs of low volume) has been associated with a bacterial etiology ( *Campylobacter*, *Salmonella*, *Shigella*, *Yersinia*). In those circumstances, antibiotic therapy should be provided at least in countries where the mortality rate is consistent or with limited healthcare facilities, according to the WHO. Alternatively, it could be considered but not necessarily given. Microbiological investigation should always be obtained in dysenteric diarrhea, but, in severe cases, empiric therapy should be started while awaiting the results. Finally, a prolonged course of diarrhea in a child who is losing weight also requires microbiological investigation and occasionally empirical antibiotic treatment ^32^. Prolonged diarrhea may be caused by a proliferation of intestinal bacteria in the proximal intestine, so-called small intestinal bacterial overgrowth (SIBO). A recent paper proposes an interesting explanation of the link among SIBO, infectious irritable bowel syndrome, and tropical sprue, all conditions that are successfully treated with antibiotics ^33^. Microbiological results may support the decision to treat with antibiotics. Figure 1 shows the criteria for consideration when deciding on antibiotic treatment for children with infectious diarrhea.

Antibiotic therapy is always recommended for culture-proven (or even suspected) *Shigella* gastroenteritis. Antibiotic therapy of shigellosis has two purposes: reducing symptoms and sterilizing the source of spreading, since humans are the only host of *Shigella*. However, effective treatment of shigellosis is complicated by the emergence of strains resistant to ampicillin, trimethoprim-sulfamethoxazole, and tetracycline ^34^. Children with non-typhoidal *Salmonella* gastroenteritis should not be treated routinely with antibiotics because treatment is not effective on symptoms and does not prevent complications; in addition, the use of antibiotics may be associated with a prolonged fecal excretion of *Salmonella* ^1^. Antibiotic therapy for *Campylobacter* gastroenteritis is recommended mainly for the dysenteric form and to reduce transmission in day-care centers and institutions. However, antibiotics are effective in reducing symptoms only if started in the early stage of the disease (within 3 days of onset).

### Host-related indications

Host-related indications include age, the finding of specific pathogens, the presence of chronic underlying diseases, immune suppression, and malnutrition (see Table 2).

***Age*.** AGE in neonates should be treated with antibiotics. Also, young infants (under 3 to 6 months of age) are candidates for antimicrobial therapy according to expert opinion, although there is no supporting evidence ^1^. In infants under 3 months of age, microbiology should always be obtained and antimicrobial treatment should be considered. If diarrhea is severe or if there are signs or clinical symptoms of general infection, or also if symptoms are worsening after 3 or more days from their onset, antibiotic therapy should be started.

***Chronic conditions.*** International guidelines state that children with underlying immune deficiency, anatomical or functional asplenia, corticosteroid or immunosuppressive therapy, cancer, inflammatory bowel disease (IBD), or achlorhydria should receive antibiotics when bacterial gastroenteritis is suspected. Although this approach appears logical, data on efficacy are lacking, the grade of evidence is weak, and there is no list of specific chronic conditions that require antibiotic therapy for diarrhea.

Selected agents are associated with immunodeficiency or other specific diseases, and the major bacterial opportunistic agent is Cd.

Cd has reached epidemic proportions, particularly in industrialized nations. Cd is a major agent of antibiotic-induced diarrhea and of severe diarrhea in children with underlying chronic conditions such as IBDs as well as oncologic diseases. Cd is also responsible for self-limiting, sporadic cases of AGE in children, although its pathogenic role is limited or questionable in children under 36 months of age because of the high frequency of carriers ^35^. Cd-induced antibiotic diarrhea often resolves by discontinuation of the antibiotic. However, hypervirulent strains may induce severe symptoms and should be treated with oral metronidazole or vancomycin ^36^. For moderate or severe disease, particularly in oncologic patients, the first-line treatment is oral metronidazole (30 mg/kg/day); oral vancomycin is reserved for resistant strains ^37, 38^. If antibiotic therapy fails, fecal transplantation remains a feasible and effective option ^37^.

Patients with IBD are at increased risk of Cd infection. An increased incidence of Cd infections in this population has been reported also in pediatric patients ^37, 39^. Nevertheless, there are substantial problems in defining the role of Cd owing to the frequent asymptomatic status. IBD patients have a higher asymptomatic Cd carriage status: as high as 8% compared to a rate of 1% in healthy subjects ^40^. Antibiotic exposure seems to be a less important factor for clinically significant Cd infections in IBD patients. The proposed mechanism of Cd infections involves an alteration of the intestinal flora ^8^. In addition, clinically, IBD exacerbations and Cd infections are similar in the IBD population, with bloody diarrhea and systemic symptoms, such as fever, malaise, anorexia, leukocytosis, hypoalbuminemia, and stool leukocytes in both conditions. Antibiotics may be useful, although in both IBD patients and oncologic patients there is a paucity of evidence to guide antibiotic choice. Metronidazole has been associated with a high rate of failure, and it may be reasonable to consider vancomycin as first-line treatment of severe cases ^41^.

However, all the international guidelines recommend microbiological examination and to start metronidazole or ciprofloxacin in IBD children with diarrhea recurrence. Again, there are no controlled studies to support this albeit reasonable strategy. Also, in children with cancer, intestinal infections are a major threat and require a comprehensive diagnostic approach ^38^.

***Immunocompromised patients.*** The major source of information on the link between incidence and severity of gastrointestinal infections and immunodeficiency is derived from children with AIDS. In 2010, a study from Kenya showed that diarrhea was more common among human immunodeficiency virus (HIV)-positive children than among HIV-negative children (321 versus 183 episodes respectively, *p*<0.01) and that diarrhea was associated with a 40% fatality rate. In addition, HIV-positive infants were significantly more likely to experience persistent diarrhea than HIV-negative infants ( *p*<0.01). Although diarrhea was more common among HIV-infected children, bacterial pathogens such as *Campylobacter* and *Shigella* were not frequent, suggesting that other pathogens (e.g. viruses, parasites, diarrheagenic *E. coli*) or other causes (e.g. malabsorption, metabolic enteritis) may be important in this population ^42^. However, *Cryptosporidium parvum* is the classical agent of diarrhea in severely immunodeficient children, and its detection is considered a hallmark of severe disease. HIV itself can act as an enteric pathogen through the production of an enterotoxic effect ^43^.

***Malnutrition.*** Children with severe acute malnutrition (SAM) who present with AGE are generally treated with broad-spectrum antibiotics, even in the absence of overt infection. The rationale is that (a) malnourished children frequently have bacterial infections (including bacteremia), (b) the diagnosis of infection in malnourished children is difficult because clinical manifestations (e.g. fever) may not be apparent, and (c) malnourished children have an increased risk of small intestinal overgrowth. However, while this approach has a rational basis, there is very little evidence of its efficacy. A study from Malawi clearly demonstrated the importance of antibiotic administration to children with SAM even without evident clinical features of infection: 2,767 children with SAM eligible for outpatient care and aged 6–59 months were randomized to 7 days of treatment with oral amoxicillin, cefdinir, or placebo. The 12-week mortality rates were 4.8% (amoxicillin), 4.1% (cefdinir), and 7.4% (placebo), with a relative mortality risk for placebo compared with amoxicillin of 1.55 (95% CI 1.07–2.24) and for placebo compared with cefdinir of 1.80 (95% CI 1.22–2.64) ^44^. SAM is associated with an increased mortality from infectious diseases, suggesting that children with SAM are severely immunologically impaired. However, the precise mechanisms underlying this relationship are unclear. Diarrhea and malnutrition are common in young children in developing countries, and malnutrition is associated with increased severity of common infections. Death of severely malnourished children is often the result of an infection. Children with AGE were significantly more likely to have malnutrition (OR=8.57; *p*<0.001), and malnutrition status was the only independent factor associated with infection (OR=8.37; *p*<0.001) ^45^. Environmental enteropathy, recently redefined as environmental enteric dysfunction, is the combined result of undernutrition, repeated infections, and environment-related toxic damages occurring in early life, requiring a comprehensive approach with anti-infective drugs, hygiene measures, and nutritional rehabilitation to prevent subsequent severe disabilities ^46^. Therefore, the management of infection should be different in malnourished versus well-nourished children, and a more aggressive antimicrobial strategy is indicated in the former.

## Choice of antimicrobial agent

In the past 10 years, new molecular diagnostic tests with a multiplex polymerase chain reaction (PCR) panel have been developed. They are faster than traditional tests, have a higher sensitivity, and have the possibility to simultaneously test a wide range of agents ^47^. Molecular diagnostics would enable the physician to initiate timely and targeted antibiotic therapy. Early empiric antibiotic therapy will remain the therapy of choice for severely affected patients.

The decision to treat a child with AGE and the choice of antimicrobial drug is challenging. There is a relatively broad pattern of pathogens according to age, location, season, vaccine policy (against rotavirus and others), and symptoms ^48, 49^. Furthermore, infections with multiple pathogens, which are common among children with diarrhea, complicate treatment. Antimicrobial resistance should also be considered in the antibiotic choice. Knowledge of the local pattern of resistance is crucial to reduce the number of failures. Antibiotic selection is based on two major considerations: the chance of obtaining microbiological results, including resistance pattern, and the severity of clinical conditions.

The WHO recommends treating all episodes of blood in the stools with antibiotics and to use ciprofloxacin as the first-line drug. Alternatives are pivmecillinam, azithromycin, and ceftriaxone ^50^. This recommendation has been confirmed, although in recent years the rates of resistance are increasing ^25^. Fluoroquinolones are often empirically used in adults, and cephalosporins are used to treat children with suspected bacterial AGE. Fluoroquinolones are effective against a wide variety of enteric infections in adults, including shigellosis, salmonellosis, typhoid fever, cholera, and *Campylobacter* infections. Like all quinolones, ciprofloxacin causes arthropathic effects in immature animals and their use has been limited in children. However, several studies have confirmed the safety of ciprofloxacin use in the pediatric age group. Because of low cost and the availability of an oral formulation, ciprofloxacin plays an important role in the treatment of childhood acute invasive diarrhea, especially in poor countries.

Often, in severe conditions, early empiric therapy is needed while awaiting the results of investigations. If clinical conditions are severe, parenteral therapy should be started soon. For parenteral therapy of diarrhea, ceftriaxone or ciprofloxacin may be considered, as both are effective against Gram-negative bacteria. In children with chronic conditions, metronidazole provides an alternative option, as it is also effective against Cd. Oral metronidazole can be considered for sequential therapy after parenteral administration. Oral metronidazole is used for prolonged diarrhea, although there is little evidence of efficacy of antibiotics ^32^.

SIBO is another indication for antibiotics. It may be difficult to diagnose, as quantitative cultures of duodenal aspirate as well as the breath hydrogen test are neither standardized nor reliable ^51^. Co-trimoxazole and metronidazole are first-line drugs ^52^. The latter is effective for bacterial agents, including Cd, as well as against *Giardia lamblia—*all agents implicated in prolonged diarrhea. Recently, rifaximin has been used in clinical (uncontrolled) trials with good results ^53^.

Co-trimoxazole is still largely used in the antimicrobial therapy of diarrhea. It has been effective in malnutrition and HIV-related enteropathy and is a major drug with multiple indications in developing countries ^54^.

In high-income countries, untargeted antibiotic therapy should be avoided. However, azithromycin is the drug of choice for treating campylobacteriosis and is also appropriate for treating shigellosis ^1^. The duration of treatment is 3–5 days.

Non-typhoidal *Salmonella* infections are common in many settings and endemic in European children. Usually, they cause mild, self-limiting gastroenteritis. However, bacteremia may be a complication—particularly in immunocompromised children, in those with sickle cell disease, and in young infants—and in those children antibiotic therapy should be considered ^55^. Recommended empiric oral treatment of non-typhoidal salmonellae includes amoxicillin, azithromycin, or co-trimoxazole and should be considered for at-risk children in relatively good clinical conditions. Parenteral therapy should be started in children with bacteremia or in those with complicated infections (focal or invasive) and includes cefotaxime or ceftriaxone at high dose (ceftriaxone 100 mg/kg/day) ^56^.

In the case of traveler’s diarrhea, antibiotic treatment is effective in reducing the duration and severity of diarrhea. Because of the high rates of resistance to ampicillin and trimethoprim-sulfamethoxazole, currently the drugs recommended include azithromycin, ciprofloxacin, and rifaximin ^57^. Rifaximin may be considered as a first-line treatment option in adults with uncomplicated traveler’s diarrhea because of its favorable efficacy, tolerability, and safety profiles ^57^.

The choice of antibiotic therapy based on etiology is summarized in Table 3.

**Table 3.** Antibiotic choice based on etiology.

Organism	Preferred therapy	Alternative agents	Efficacy
*Campylobacter jejuni*	Azithromycin	Ciprofloxacin, vancomycin	Proven if started within 3 days of symptom onset
*Clostridium difficile*	Metronidazole	Vancomycin	Proven in severe cases
Non-typhoidal Salmonella	Amoxicillin or ceftriaxone	Trimethoprim- sulfamethoxazole	Proven in children with toxic status, in children under 3 months of age, in at-risk children, and if systemic or focal infections
*Salmonella typhi*	Third-generation cephalosporins	Chloramphenicol	Proven
*Shigella*	Azithromycin, ceftriaxone	Cefixime, ciprofloxacin	Proven
*Yersinia*	Trimethoprim- sulfamethoxazole	Ceftriaxone	Proven in severe disease or bacteremia
*Vibrio cholerae*	Azithromycin	Doxycycline (>8 years), ciprofloxacin	Reduces duration by 50% and shedding
ETEC	Azithromycin (only for traveler’s diarrhea)	Trimethoprim- sulfamethoxazole	To be considered in selected cases

ETEC, enterotoxigenic *Escherichia coli*.

## Conclusions

Rehydration is the key treatment for AGE, and active treatment of diarrhea with probiotics or diosmectite should always be considered, independent of etiology. Antibiotics are generally not necessary and can even be harmful in children, but they should be given in selected circumstances. There are three distinct sets of criteria that should be carefully considered: clinical conditions, host-related factors, and setting. When there is a potential indication for antibiotics, microbiological investigations should always be obtained prior to the start of therapy. Empiric antibiotic therapy should be started soon after specimen collection in infants and children in severe conditions. Co-trimoxazole and metronidazole are to be considered for oral administration. Azithromycin and rifaximin may also be used, based on local consideration or if signs of colitis are observed. Ceftriaxone, metronidazole, and ciprofloxacin may be considered in children with systemic and invasive diseases. Young infants, children with chronic conditions, and those in a toxic state or with signs of systemic infection should be considered at risk of systemic infections, and oral or parenteral antibiotic therapy may be indicated. If mild symptoms are present and close observation is feasible, it may be better to wait for microbiological results. Antibiotic therapy in specific settings is also indicated if spreading is an issue. Traveler’s diarrhea may require antibiotic therapy. The choice of specific antibiotic should be based on etiology and local resistance pattern.

In conclusion, while it is important to reduce the use of unnecessary antibiotics, there are circumstances in which these drugs are needed and are potentially life-saving. However, their use is far from being supported by evidence and requires careful consideration of clinical and epidemiological issues.

## Abbreviations

AGE, acute gastroenteritis; EAEC, enteroaggregative *Escherichia coli;* EPEC, enteropathogenic *Escherichia coli*; ETEC, enterotoxigenic *Esherichia coli;* Cd, *Clostridium difficile*; HIV, human immunodeficiency virus; IBD, inflammatory bowel disease; SAM, severe acute malnutrition; SIBO, small intestinal bacterial overgrowth; WHO, World Health Organization.
